# Phenotype and Metabolic Disorders in Polycystic Ovary Syndrome

**DOI:** 10.5402/2012/569862

**Published:** 2012-02-29

**Authors:** Olgierd Głuszak, Urszula Stopińska-Głuszak, Piotr Glinicki, Renata Kapuścińska, Hanna Snochowska, Wojciech Zgliczyński, Romuald Dębski

**Affiliations:** ^1^The Medical Centre of Postgraduate Education, ul. Marymoncka 99/103, 01-813 Warsaw, Poland; ^2^Department of Endocrinology CMKP, Bielański Hospital, ul. Cegłowska 80, 01–809 Warsaw, Poland

## Abstract

The polycystic ovary syndrome (PCOS) is one of the most frequent endocrinopathies in women. Its incidence is assessed at 6–8% of the female population in the reproductive age. It is characterised by oligomenorrhea (Oligo), hyperandrogenism (HA), and the presence of polycystic ovaries (PCOs). Carbohydrate and lipid metabolism is being disturbed in many women with PCOS. The pathogenesis of PCOS is still unexplained. Following the main criteria of diagnosis (Rotterdam Consensus 2003), Dewailly, Welt and Pehlivanov divided the patients with PCOS into 4 phenotype groups: A, B, C, and D. In our studies of 93 patients with PCOS, we found (1) the most frequent appearance (60,2%) of the phenotype A [Oligo + HA + PCO]; (2) an increased androstenedione concentration in a group with HA (A, B, C); (3) an increased HOMA-*β* and insulin concentration after 30 min an oral 75 g glucose tolerance test (OGTT) in a group of obese women with BMI > 30 kg/m^2^; (4) high levels of total testosterone, total cholesterol, and LDL cholesterol concentrations in a group A with classic phenotype of PCOS: Oligo + HA + PCO—increasing the risk of development of cardiovascular diseases, type 2 diabetes, or metabolic syndrome. The average androstenedione concentrations could be a good diagnostic and prognostic parameter.

## 1. Introduction

Polycystic ovary syndrome (PCOS) is one of the most common female endocrine disorders affecting approximately 6–8% of women in the reproductive age. It is one of the main causes of infertility resulting from chronic anovulation. This syndrome was first described in 1935 by Stein-Leventhal who observed among some patients menstrual disorders and polycystic ovaries (the “billiard ball” sign on ultrasound examination) [1]. The most common irregularities of PCOS include elevated serum levels of free testosterone (T), androstenedione, dehydroepiandrosterone sulfate (DHEAS), excessive amount of luteinizing hormone (LH), elevated LH/FSH ratio, increase in LH peak pulse frequency and its response to GnRH (Gonadotropin-releasing hormone), and change in LH pulse frequency. Insulin resistance, obesity, dyslipidemia, elevated laboratory findings associated with inflammation, high blood pressure, and increased risk of cardiovascular diseases are the common symptoms of PCOS. Albeit the research trials are broad, the pathogenesis remains uncertain.

### 1.1. Criteria for Defining PCOS

In 1990, a consensus workshop sponsored by the NIH/NICHD [2, 3], PCOS was defined as: menstrual disorders and hyperandrogenism after exclusion of other endocrine disorders such as hyperprolactinemia, thyroid gland disorders, and congenital adrenal hyperplasia. At the conference in Hamburg [2], the additional diagnostic criteria for PCOS were added: acne, hirsutism, elevated blood levels of androgens, and increased insulin resistance. Today's definition of PCOS was defined by a consensus workshop sponsored by ESHRE/ASRM in Rotterdam in May 2003 [4]. It includes

1. menstrual disorders (oligoovulation/anovulation),
2. clinical and/or biochemical evidence of hyperandrogenism,
3. polycystic ovaries on ultrasound examination (at least 10 follicles 2–9 mm in size or volume of the ovary greater than 10 mL).

In 2005, Azziz introduced modification of NIH criteria for PCOS regarding androgen excess and ovaries dysfunction (irregularity or absence of menses accompanied with visualization of polycystic ovaries) [5].

### 1.2. Phenotype

Regarding the Rotterdam criteria for PCOS [4, 6], both endocrinal and clinical, we distinguish 4 different phenotypes of the syndrome, as shown in Table 1:

- Oligo + HA + PCO,
- Oligo + HA,
- HA + PCO,
- Oligo + PCO.

In 2005, Chang et al. [5] proposed another division of the phenotype into 3 groups (See Table 2).

- Oligo + HA biochemical + hirsutism,
- Oligo + HA biochemical,
- Oligo + hirsutism.

In the similar manner, Hassa et al. [7] introduced resembling division of PCOS's phenotype into 3 groups (See Table 2).

(I′) Oligo + HA clinical and biochemical,
(II′) Oligo + HA biochemical,
(III′) Oligo + HA clinical.

### 1.3. Objective

The objective of the study was to define hormonal, biochemical, and metabolic abnormalities among women with PCOS arising from a group of 4 standard phenotypes and then to identify coexistence of endocrine and biochemical abnormalities in 4 groups of patients who are at increased risk of metabolic diseases.

## 2. Material and Methods

We identified 93 women who met the current ESHRE/ASRM criteria for PCOS [4, 6] and evaluated their hormonal and biochemical profiles. The physical examination included measurements of circumferences of their waist and hips and calculation of the body mass index (BMI). We also evaluated the extent of hyperandrogenism (hirsutism—using the Ferriman Gallwey hirsutism evaluation system [8]; acne—using the subjective 0–10 scale) and used both: the insulin resistance index HOMA-IR with HOMA-*β*-cell index (assessing beta-cell function) and QUICK (Quantitative Insulin Sensitivity Check) index, regarding insulin resistance [5]:

(1) $$\begin{array}{c} \text{HOMA-IR}\;\text{index}\;=\;\frac{\left(\text{Io}\;\times \;\text{Go}\right)}{22,5},\\ \text{HOMA-}\beta \text{-cell}\;\text{index}\;=\;\frac{\left(20\;\times \;\text{Io}\right)}{\left(\left(\text{Go}\;\times \;0,05551\right)-3,5\right)},\;\\ \text{QUICK}\;\text{index}\;=\;\frac{1}{\left[\log\;\left(\text{Io}\right)+\;\log\;\left(\text{Go}\right)\right]}. \end{array}$$

The above formulas refer to the fasting levels of glucose [mg/dL] (Go) and insulin [*μ*U/mL] (Io).

The presented results were prepared by classifying the women into four groups (Rotterdam Study) according to their phenotype (A, B, C, D) [4, 6]. The obtained results were analyzed in terms of these 4 groups and additionally in terms of three basic groups that include menstrual disorders (oligoovulation/anovulation), clinical and/or biochemical evidence of hyperandrogenism, and polycystic ovaries on ultrasound examination.

In each patient, we determined the serum levels of high-sensitivity C-reactive protein (hsCRP), androgens: testosterone, androstenedione, dehydroepiandrosterone sulfate, estradiol (E2), 17-hydroxyprogesterone (17-HOP), LH, FSH (follicle-stimulating hormone), sex hormone binding globulin (SHBG), lipids, aminotransferases, glucose and insulin fasting and after ingestion of 75 g glucose (measurements in 30th, 60th, and 120th min after 75 g glucose ingestion). An ultrasound examination of the reproductive system, in particular the ovaries, was performed on each patient. The examinations were carried out during the first phase of the menstrual cycle (1st–10th day of the cycle) in all women with PCOS, except for the patients who had been taking medications for any reason during the last 3 months.

In the recruiting process, we had to exclude the patients with coexisting thyroid gland disorders, diabetes mellitus, ovarian and adrenal tumors, congenital adrenal hyperplasia, and other important pathologies. We determined the serum levels of thyroid-stimulating hormone (TSH), adrenocorticotropic hormone (ACTH), 17-hydroxyprogesterone (17-HOP), and metoclopramide-stimulated prolactin (PRL) serum levels. We also performed the ultrasound examination both of the thyroid gland and abdominal cavity, chest radiography, collection of 24-hour urine steroids derivatives: 17-hydroxycorticosteroids (17-OHCS) and 17-ketosteroids (17-KS) in terms of normal production and suppression with small amount of Dexamethasone (4 × 0,5 mg) for two days.

Diagnostic criteria for diabetes mellitus were based on the fasting serum glucose level and serum glucose level in the oral glucose tolerance test (OGTT) after a 75 g glucose load. The serum level of 17-OHP above 5 mg/dL was highly suspicious for the congenital adrenal hyperplasia. The tests were repeated for the results above 3.5 mg/dL.

The diagnosis of the thyroid gland disorders was established by determining TSH, free thyroxine (FT4), and antithyroid autoantibodies. The diagnosis of the hirsutism was established by gathering at least six points using the Ferriman Gallwey hirsutism evaluation system. Acne was diagnosed in these patients who received four or more points using the subjective 0–10 scale, in which we assessed face, back, and other body parts (max. 20 points). Blood was collected from the fasting patients in the morning. They were between 1st and 10th day of their menstrual cycle. The hormonal blood tests were performed by using both radioimmunoassay (RIA) and IRMA methods and immunochemiluminescent method with the Immulite 2000 (Dpc, Los Angeles, Ca) device at the Endocrine Clinic of Medical Centre of Postgraduate Education (CMKP).

Normality of analyzed variables was tested with Saphiro-Wilk test. Comparisons between groups were performed by ANOVA or Kruskal-Wallis tests. To compare two independent groups of observations, Mann-Whitney *U* test has been used. The significance level alpha was chosen at 0,05. The statistical analysis was performed using Statistica 9.0 PL.

## 3. Results

The most common phenotype was phenotype A (60,2%, *P* < 0,05). Phenotype A met all 3 current criteria for the PCOS. Its prevalence was similar to the numbers quoted by other authors (58,6%–71%) [9–11]. The other three phenotypical groups appeared significantly more rarely, namely, C (18,3%), B (16,1%), and D (5,4%), and, according to three other authors, their prevalence was different (Table 3).

The prevalence of the PCOS in three phenotypical groups in our trial was comparable to the results presented by Chang and Hassa et al. [5, 7]. The most common patients were those who met all three diagnostic criteria for the PCOS, although there were some differences in the percentages among the cited authors (I-69,7%, I′-84,85%). The differences could have resulted from the numeral differences among test populations and the diagnostic criterion for the hyperandrogenism affecting the prevalence of acne.

The presented trial did not show the statistical significance in the differences such as patients' average age, weight, height, circumference of waist and hips, waist to hip ratio, and body mass index (*P* > 0.05), among the four described phenotypical groups (A, B, C, D). Although there were significant statistical differences regarding the average ovary volume, prevalence of hirsutism, acne, and the length of the menstrual cycle (*P* < 0,05), which was in compliance with the recruitment and classification of the individuals to the specific phenotypical groups (Table 4).

The highest serum levels of testosterone and dehydroepiandrosterone sulfate (DHEAS) were observed in women who presented clinical evidence of hyperandrogenism (groups A, B, and C) (Table 5). Analyzing each group with the hyperandrogenism alone, the levels of serum testosterone were statistically higher (*P* < 0,04) in the group A, comparing to the group D. Despite the observed higher levels of serum testosterone in the group B and C, the B/D correlation was not statistically significant. However, there were observed statistically higher levels (*P* < 0,05) of serum testosterone and DHEAS in the group C in comparison to the group without the signs of hyperandrogenism (group D). The levels of androstenedione were very clearly elevated (*P* < 0,004) in all three groups with hyperandrogenism (A, B, C), when comparing them to the group D—without the signs of hyperandrogenism. Similarly, when we compare each group with the hyperandrogenism, the levels of androstenedione were statistically significantly higher (A/D *P* < 0,005; B/D *P* < 0,013; C/D *P* < 0,004). Although the highest levels of androstenedione were present in the phenotypical group B (mean 525,17 ng/dL ±146,08 ng/dL) which presents no morphological signs of polycystic ovaries on ultrasound examination, the androstenedione B group levels were significantly higher than in the group C (*P* < 0,05).

Similarly to androstenedione, the levels of 17-HOP were significantly higher (*P* < 0,03) in all three groups with hyperandrogenism (A, B, C), when compared to the group D (Table 5). A resembling, statistical correlation was observed when the levels of 17-HOP in the group A and D were compared (*P* < 0,02).

The higher levels of LH and estradiol (E2) were observed in the group A in comparison to the group C (without menstrual disorders) and the average value of the parameters differed in the statistical significance (*P* < 0,05). The highest average levels of LH and E2 were detected in the group B (without morphological signs of PCOS on ultrasound examination), although these results were not significant, probably because of the greater variability of these parameters in both groups.

The elevated values of the LH/FSH ratio were noticed among women in the group A and C when compared to the group D (without hyperandrogenism). Because of the small number of women in the group D, this remark was not statistically proven and has been left as the object of further investigation.

There were no statistical significant differences between the presented phenotypical groups B, C, and D regarding total cholesterol, HDL-cholesterol, LDL-cholesterol, or triglyceride levels. However, the levels of total cholesterol (184, 23 ± 33,86 versus 165, 12 ± 23,50 mg/dL; *P* < 0,05) and LDL cholesterol (103, 14 ± 29,35 versus 87, 02 ± 21,40 mg/dL; *P* = 0,04) were significantly higher in the group A comparing to the three other groups.

The greatest levels of 17-KS in urine (steroid metabolites) were measured in the group C without menstrual disorders (17, 28 ± 4, 2 mg/dL, norm: 4,2–16.3 mg/dL). The difference between the levels of 17-KS in urine in the group C and the groups A, B, and D was of the statistical significance (*P* < 0,05).

The higher levels of 17-OHCS in urine were observed in the groups B and D, in which the obese women (BMI 30 kg/m^2^ and greater) predominated (*P* < 0,003), in comparison to the groups A and C (in which there was a slight majority of slim women; BMI <27 kg/m^2^).

In the observed population, the lowest levels of insulin in 30th minute after 75 g glucose load (OGTT) were seen among women in the group A and the levels were statistically lower (*P* < 0,001) in comparison to the other three groups (B, C and D), where there was the biggest percentage of obese women (BMI > 30 kg/m^2^). Similar results were noticed regarding fasting insulin, but they could not be confirmed statistically (Table 6).

The highest levels of insulin in 30th minute (94, 44 ± 50, 27 uU/mL) were present in the groups B and D with the biggest percentage of the obese women (*P* < 0,0005) when compared to the groups A and C (51, 66 ± 25, 84 uU/mL). The index HOMA-*β* that assesses beta-cell function was statistically higher in the groups B and D in comparison to the groups A (*P* < 0,05) and A + C (*P* < 0,01). However, the indexes HOMA-IR and QUICK index showed no statistical significance.

## 4. Discussion

The presented division into four standard phenotypical groups (ESHRE/ASRM) significantly emphasizes the existing differences in PCOS, which is not a homogenous disorder but the unit of three coexisting elements: menstrual disorder, hyperandrogenism, and presence of the polycystic ovaries [4, 6]. Moreover, we all observed disorders in lipid and carbohydrate metabolism. Our analysis of 93 women with the PCOS showed that the elevated levels of insulin in the 30th minute after 75 g glucose load in the group Oligo + HA (B) in comparison to the average insulin level in the 30th min in the group of women who do not present the polycystic ovaries on the ultrasound examination (A, C, and D) may be related to slightly increased prevalence of obesity and hypertriglyceridemia among these individuals.

Similar observations were noticed regarding beta-cell function index (HOMA-*β*), which reached the highest levels in the groups B and D, when compared to the groups A and AC, which seems to be connected with the obesity prevalence.

The levels of indexes HOMA-IR and QUICK, regarding the insulin resistance, showed no statistical significance in the analyzed groups, although there was some upward tendency of the HOMA-IR index accompanied by decrease of the QUICK index in the groups B and D, with the elevated number of obese women. This data requires further investigation.

In the analyzed groups, there were no statistically significant differences among the presented phenotypical groups B, C, and D when it comes to total cholesterol, HDL cholesterol, LDL cholesterol, or triglycerides, although the levels of total cholesterol and LDL cholesterol were commonly higher than normal, especially in the phenotypical group A (21 of 56 individuals), which is suggestive of intensification of dysregulation and increased risk of cardiovascular and metabolic diseases among women with complete phenotype of PCOS. The major amount of abnormalities in the phenotypical group A was also seen by other authors [9–11].

The androstenedione levels were clearly elevated in all three groups with hyperandrogenism (A, B, C), when compared to the group D without the signs of hyperandrogenism. When we compared particular groups with hyperandrogenism, the levels of androstenedione were statistically significantly higher. That is why the measurement of the androstenedione levels seems to be a crucial diagnostic and predictive factor among women with menstrual disorders or present polycystic ovaries of ultrasound examination. The elevated serum levels of androstenedione in the group B, with no sign on polycystic ovaries on ultrasound examination, in comparison to the phenotypical group C, require further investigation.

The higher than normal levels of 17-KS in urine in group C (without menstrual disorders) could be a sign of increased role of the ovaries in the androgen production in this group, when compared to the other groups with such disorders (A, B, D). Nevertheless, further studies are necessary.

The greatest levels of 17-OHCS in urine were observed in the groups B and D (in which the obese women with BMI > 30 kg/m^2^ predominated) when compared to the groups A and C. This might indicate the greater role of the adrenal gland in the androgen production among obese women and the increased risk of the insulin resistance in the group of obese individuals with PCOS.

## 5. Conclusions

In the performed study regarding 93 women with the PCOS, the following correlations were determined.

1. The major prevalence of the phenotypical group A, which is similar to the result presented by the other authors.
2. The increased level of androstenedione strongly correlates with the clinical degree of hyperandrogenism. It seems that androstenedione could be a crucial diagnostic and predictive factor among women with menstrual disorders or presence of polycystic ovaries on ultrasound examination.
3. The elevated levels of the indexes that correlate with the degree of insulin resistance such as the beta-cell function index (HOMA-*β*) or insulin level in 30th min, after 75 g glucose load, are met more often among obese women with BMI > 30 kg/m^2^, rather than in slim individuals, without coexisting hyperandrogenism or presence of polycystic ovaries on ultrasound examination.
4. In the phenotypical group A, there were noticed elevated levels of total testosterone, androstenedione, and significantly higher levels of total cholesterol and LDL cholesterol. It may point out the increased risk of cardiovascular and metabolic diseases. Because of the small number of women in the investigated group and too short a period on the trial, this thesis needs to be the subject of further studies.

The presented division into four phenotypical groups and the observed correlations contribute to the greater understanding of the core of the polycystic ovary syndrome and to the better recognition of its pathogenesis.

## Figures and Tables

**Table 1:** 

Phenotypical groups:	A	B	C	D
Menstrual disorders (Oligo)	+	+		+
Clinical and/or biochemical hyperandrogenism (HA)	+	+	+	
Polycystic ovaries (PCO)	+		+	+

**Table 2:** 

	I	II	III
Menstrual disorders (Oligo)	+	+	+
Biochemical hyperandrogenism (HA-b)	+	+	
Clinical hyperandrogenism (HA-c)	+		+

**Table 3:** Prevalence of 4 phenotypical groups (A, B, C, D) in the PCOS.

		Prevalence of PCOS		
		Dewailly et al.—France [9]	Welt et al.—Boston/Iceland [10]	Pehlivanov and Orbetzova—Bulgary [11]
Group (no.)	**93**	406	418	70
Phenotype A	**60,2%**	60,6%	71%	58,6%
Phenotype B	**16,1%**	6,7%	2%	11,4%
Phenotype C	**18,3%**	16,5%	18%	10,0%
Phenotype D	**5,4%**	16,3%	9%	20,0%

**Table 4:** Features of the individuals with PCOS, classified to 4 phenotypical groups (A, B, C, D).

Phenotypical group	A	B	C	D
	Oligo + HA + PCO	Oligo + HA	HA + PCO	Oligo + PCO
Number of individuals	**56**	**15**	**17**	**5**
Average age (years)	24, 3 ± 4,5	21, 9 ± 4,8	24, 6 ± 5,1	24, 0 ± 7,0
Age 15–20 years	**6 **(10,7%)	** 6 **(40%)	**2 **(11,8%)	** 2 **(40%)
Age 20–25 years	**35 **(62,5%)	**7 **(46,7%)	**11 **(64,7%)	** 1 **(20%)
Age 25–30 years	**9 **(16,1%)	**0**	**2 **(11,8%)	** 2 **(40%)
Age > 30 years	**6 **(10,7%)	**2 **(13,3%)	**2 **(11,8%)	**0**
BMI (kg/m^2^)	26, 22 ± 7,1	28, 95 ± 7,2	26, 12 ± 6,5	28, 38 ± 8,6
Weight (kg)	71, 6 ± 19,3	82, 1 ± 22,3	75, 2 ± 19,9	80, 8 ± 17,7
Height (cm)	165, 3 ± 6,9	168 ± 5,15	169, 6 ± 4,0	169, 8 ± 7,4
Circumference of waist (cm)	81 ± 14,6	86, 4 ± 14,0	81, 1 ± 13,3	87, 8 ± 13,08
Circumference of hip (cm)	103 ± 12,9	110, 1 ± 16,5	104, 8 ± 11,7	107 ± 6,36
Circum. waist/hip index	0, 78 ± 0,06	0, 78 ± 0,05	0, 77 ± 0,06	0, 82 ± 0,07
Average ovary volume (mL)	14, 08 ± 4,01	6, 9 ± 1,93	13, 31 ± 3,61	11, 63 ± 2,15
Hirsutism	**43 **(76,8%)	**11 **(73,3%)	**12 **(70,6%)	**0**
Acne	**32 **(57,1%)	**13 **(86,7%)	**11 **(64,7%)	**0**
Average cycle length (days)	91, 7 ± 44,82	35 ± 6,0	30, 8 ± 1,12	35, 5 ± 0,7
Cycle length 27–34 dni (%)	** 0**	**0**	** 17 **(100%)	**0**
Cycle length 35–44 dni (%)	** 11 **(19,6%)	** 9 **(60%)	**0**	** 4 **(80%)
Cycle length 45–90 dni (%)	** 22 **(39,3%)	** 4 **(26,7%)	**0**	** 4 **(20%)
Cycle length > 90 dni (%)	** 24 **(41,1%)	** 2 **(13,3%)	**0**	**0**

Each value represents mean ± standard error of mean or number of cases (percentage).

**Table 5:** The serum levels of the hormones in women with POCS divided into 4 phenotypical groups (A, B, C, D).

Type phenotype	A	B	C	D
	Oligo + HA + PCO	Oligo + HA	HA + PCO	Oligo + PCO
Testosterone (ng/mL)	0, 88 ± 0,31	0, 78 ± 0,15	0, 76 ± 0,3	0, 55 ± 0,1
SDHEA (ng/mL)	3566, 84 ± 1203,61	3568, 33 ± 1481,27	4327, 75 ± 1514,1	2161, 00 ± 974,4
Androstenedione (ng/dL)	485, 28 ± 146,60	525, 17 ± 146,08	395, 25 ± 67,7	192, 00 ± 103,2
17-hydroxyprogesterone (ng/mL)	2, 04 ± 0,82	1, 78 ± 0,74	1, 74 ± 0,9	0, 75 ± 0,5
Estradiol (pg/mL)	47, 77 ± 14,30	54, 9 ± 30,5	37, 63 ± 11,9	39, 25 ± 0,6
LH (U/L)	9, 74 ± 4,60	11, 30 ± 11,6	6, 10 ± 3,3	4, 99 ± 2,3
FSH (U/L)	6, 64 ± 1,64	6, 45 ± 2,73	6, 10 ± 1,3	5, 94 ± 1,7
LH/FSH	1, 55 ± 0,83	1, 55 ± 0,83	1, 05 ± 0,7	0, 93 ± 0,6
SHBG (nmol/L)	47, 60 ± 32,59	39, 5 ± 29,4	44, 29 ± 24,3	39, 00 ± 10,1
Prolactin (*μ*g/L)	15, 08 ± 7,58	17, 28 ± 6,47	18, 43 ± 5,3	25, 50 ± 10,6
TSH (*μ*IU/mL)	2, 08 ± 0,99	2, 39 ± 1,57	1, 26 ± 0,7	1, 60 ± 0,6
FT4 (pmol/L)	17, 80 ± 1,70	17, 2 ± 2,62	19, 68 ± 3,1	15, 80 ± 2,0
ACTH (pg/mL)	25, 08 ± 11,37	29, 0 ± 15,1	47, 88 ± 17,7	29, 00 ± 8,5
Cortisol (*μ*g%)	15, 21 ± 3,72	18, 73 ± 7,99	20, 93 ± 5,5	18, 45 ± 0,9
17-hydroxycorticosteroids (mg/dL)	5, 23 ± 1,71	7, 04 ± 1,59	5, 54 ± 1,6	7, 40 ± 1,3
17-ketosteroids (mg/dL)	16, 45 ± 4,94	17, 28 ± 3,56	20, 78 ± 4,2	18, 85 ± 8,3

Mean ± standard error of mean.

**Table 6:** Risk of metabolic disorders in women with PCOS divided into 4 phenotypical groups (A, B, C, D) based on biochemical, hormonal and proper indexes.

Phenotypical group	A	B	C	D
	Oligo + HA + PCO	Oligo + HA	HA + PCO	Oligo + PCO
Fasting insulin (uU/mL)	7, 67 ± 5,49	9, 71 ± 4,39	8, 25 ± 3,33	8, 50 ± 6,36
Insulin after 30 min of OGTT	47, 59 ± 24,20	97, 43 ± 57,08	64, 88 ± 28,19	84, 00 ± 19,80
Insulin after 60 min of OGTT	61, 04 ± 46,02	71, 29 ± 29,12	51, 88 ± 42,31	97, 00 ± 9,90
Insulin after 120 min of OGTT	43, 22 ± 43,75	37, 14 ± 29,62	22, 88 ± 13,24	76, 50 ± 33,23
Fasting Glucose (mg/dL)	87, 68 ± 11,94	82, 37 ± 10,45	84, 40 ± 8,93	80, 00 ± 7,07
Glucose in 30 min of OGTT	142, 70 ± 20,38	132, 40 ± 26,20	120, 10 ± 32,70	134, 50 ± 7,78
Glucose in 60 min of OGTT	129, 65 ± 47,24	107, 17 ± 13,19	94, 68 ± 35,11	136, 00 ± 11,31
Glucose in 120 min of OGTT	105, 38 ± 38,53	92, 67 ± 39,22	86, 93 ± 16,90	114, 00 ± 7,07
HOMA-IR index	1, 77 ± 1,49	2, 04 ± 0,93	1, 78 ± 0,80	1, 73 ± 1,40
HOMA-*β* index	110, 89 ± 55,23	167, 14 ± 80,71	128, 78 ± 34,25	167, 01 ± 65,61
QUICK index	0, 38 ± 0,05	0, 35 ± 0,03	0, 36 ± 0,02	0, 37 ± 0,05
Peptide C (ng/mL)	2, 09 ± 0,86	2, 42 ± 1,05	1, 58 ± 0,87	2, 84 ± 0,20
HsCRP (mg/L)	2, 00 ± 1,81	1, 76 ± 1,56	1, 50 ± 1,08	2, 59 ± 0,24
Cholesterol (mg/dL)	184, 23 ± 33,86	169, 67 ± 20,03	166, 70 ± 12,28	159, 00 ± 38,18
HDL (mg/dL)	61, 74 ± 17,60	64, 24 ± 29,77	59, 81 ± 15,59	59, 90 ± 1,98
LDL (mg/dL)	103, 14 ± 29,35	87, 64 ± 26,14	92, 43 ± 14,02	81, 00 ± 24,04
Trigliceryde (mg/dL)	96, 64 ± 56,78	107, 13 ± 65,56	80, 33 ± 36,73	92, 00 ± 62,23
BMI (kg/m^2^)	26, 22 ± 7,1	28, 95 ± 7,2	26, 12 ± 6,5	28, 38 ± 8,6
BMI < 20%	**15,38%**	**14,29%**	**12,5%**	**0**
BMI 20–25%	**34,62%**	**14,29%**	**50,0%**	**50,0%**
BMI 25–30%	**26,92%**	**28,57%**	**12,5%**	**0**
BMI > 30%	**23,08%**	**42,86%**	**25,0%**	**50,0%**
